# Comprehensive characterization of stemness-related lncRNAs in triple-negative breast cancer identified a novel prognostic signature related to treatment outcomes, immune landscape analysis and therapeutic guidance: a silico analysis with in vivo experiments

**DOI:** 10.1186/s12967-024-05237-0

**Published:** 2024-05-04

**Authors:** Min Zhang, Fangxu Zhang, Jianfeng Wang, Qian Liang, Weibing Zhou, Jian Liu

**Affiliations:** 1 Xiangya Hospital, Central South University, Changsha, 41000 Hunan People’s Republic of China; 2 Department of General Surgery, The Fourth People’s Hospital of Jinan, Jinan, 250000 Shandong People’s Republic of China; 3 Department of Gastrointestinal Surgery, 970 Hospital of the PLA Joint Logistic Support Force, Yantai, 264000 Shandong People’s Republic of China; 4 Department of Pathology, University of Texas Southwestern Medical Center, Dallas, TX 75390 USA; 5 Department of Oncology, Xiangya Hospital, Central South University, Changsha, 41000 Hunan People’s Republic of China; 6 Department of Otolaryngology-Head and Neck Surgery, QingPu Branch of Zhongshan Hospital Affiliated to Fudan University, Shanghai, 201700 People’s Republic of China

**Keywords:** Triple-negative breast cancer, LncRNAs, Cancer stem cells, Prognostic model, Personalized treatment, Immune microenvironment

## Abstract

**Background:**

Cancer stem cells (CSCs) and long non-coding RNAs (lncRNAs) are known to play a crucial role in the growth, migration, recurrence, and drug resistance of tumor cells, particularly in triple-negative breast cancer (TNBC). This study aims to investigate stemness-related lncRNAs (SRlncRNAs) as potential prognostic indicators for TNBC patients.

**Methods:**

Utilizing RNA sequencing data and corresponding clinical information from the TCGA database, and employing Weighted Gene Co-expression Network Analysis (WGCNA) on TNBC mRNAsi sourced from an online database, stemness-related genes (SRGs) and SRlncRNAs were identified. A prognostic model was developed using univariate Cox and LASSO-Cox analysis based on SRlncRNAs. The performance of the model was evaluated using Kaplan–Meier analysis, ROC curves, and ROC-AUC. Additionally, the study delved into the underlying signaling pathways and immune status associated with the divergent prognoses of TNBC patients.

**Results:**

The research identified a signature of six SRlncRNAs (AC245100.6, LINC02511, AC092431.1, FRGCA, EMSLR, and MIR193BHG) for TNBC. Risk scores derived from this signature were found to correlate with the abundance of plasma cells. Furthermore, the nominated chemotherapy drugs for TNBC exhibited considerable variability between different risk score groups. RT-qPCR validation confirmed abnormal expression patterns of these SRlncRNAs in TNBC stem cells, affirming the potential of the SRlncRNAs signature as a prognostic biomarker.

**Conclusion:**

The identified signature not only demonstrates predictive power in terms of patient outcomes but also provides insights into the underlying biology, signaling pathways, and immune status associated with TNBC prognosis. The findings suggest the possibility of guiding personalized treatments, including immune checkpoint gene therapy and chemotherapy strategies, based on the risk scores derived from the SRlncRNA signature. Overall, this research contributes valuable knowledge towards advancing precision medicine in the context of TNBC.

## Introduction

Breast cancer stands as the foremost malignancy affecting women, representing a significant threat to their well-being [1–4]. Within this realm, triple-negative breast cancer (TNBC) poses a distinct clinical challenge due to its unique molecular characteristics, marked by limited treatment options, dismal prognosis, and elevated mortality rates [5–7]. Hence, there arises an urgent imperative to direct attention towards targeted exploration and comprehensive mechanistic studies centered around key tumor makers in clinical settings. Cancer stem cells (CSCs), as tumor-initiating cells, play a pivotal role in TNBC's pronounced heterogeneity, propensity for metastasis, and unfavorable prognosis. Consequently, efforts aimed at targeting their development and unraveling their mechanisms emerge as focal points in the realms of TNBC diagnostics and therapeutic investigation [8–10].

Long non-coding RNAs (lncRNAs), despite their lack of coding capacity [11–14], play significant roles in various cellular processes, including the regulation of gene expression, facilitation of subcellular transport, modulation of protein degradation, and promotion of organelle biogenesis [15]. Research indicates that abnormal expression of lncRNAs significantly influences TNBC cell behavior, impacting proliferation, migration, metastasis, and tumorigenicity [16–18]. At the transcriptional level, lncRNA regulation of gene expression was investigated using data from The Cancer Genome Atlas (TCGA). The analysis revealed that the lncRNA MIR100HG was significantly overexpressed in TNBC but not in other cancer types. Furthermore, elevated levels of lncRNA MIR100HG in TNBC patients were correlated with a poor prognosis [19, 20]. At the post-transcriptional level, lncRNAs can regulate gene expression. In TNBC, high expression of lncRNAs can competitively bind with miRNAs, acting as sponges to suppress miRNA function and promote cancer progression [21]. LncRNAs also regulate protein stability at the post-transcriptional level, thereby promoting TNBC progression [22].

Current understanding suggests that lncRNAs play a crucial role in regulating numerous biological processes of CSCs by modulating the expression of vital transcription factors responsible for stem cell functions [23], lncRNA involvement in cancer stem cell function and epithelial-mesenchymal transitions [24], LncRNA PKMYT1AR promotes cancer stem cell maintenance via activating Wnt signaling pathway [25], LncRNA LINC01315 silencing modulates cancer stem cell properties and epithelial-to-mesenchymal transition [26]. The study finding that LncRNA PART1 promotes proliferation and migration, is associated with CSCs, and alters the miRNA landscape in TNBC [27]; however, the role of stemness-related lncRNAs (SRlncRNAs) remains unclear in TNBC.

In this study, we employed WGCNA to examine the correlation between genes and mRNA expression levels, identifying stemness-related genes (SRGs). Consequently, we identified six SRlncRNAs and developed a prognostic model. Subsequently, we conducted gene set enrichment analysis (GSEA), immunoinfiltration analysis, and chemotherapy drug sensitivity analysis. Furthermore, we validated the predictive capacity of the model by inducing stemness expression in vitro in triple-negative breast cancer cells. This comprehensive approach aimed to unravel the underlying mechanisms governed by SRlncRNAs in TNBC.

## Materials and methods

**Fig. 1:**
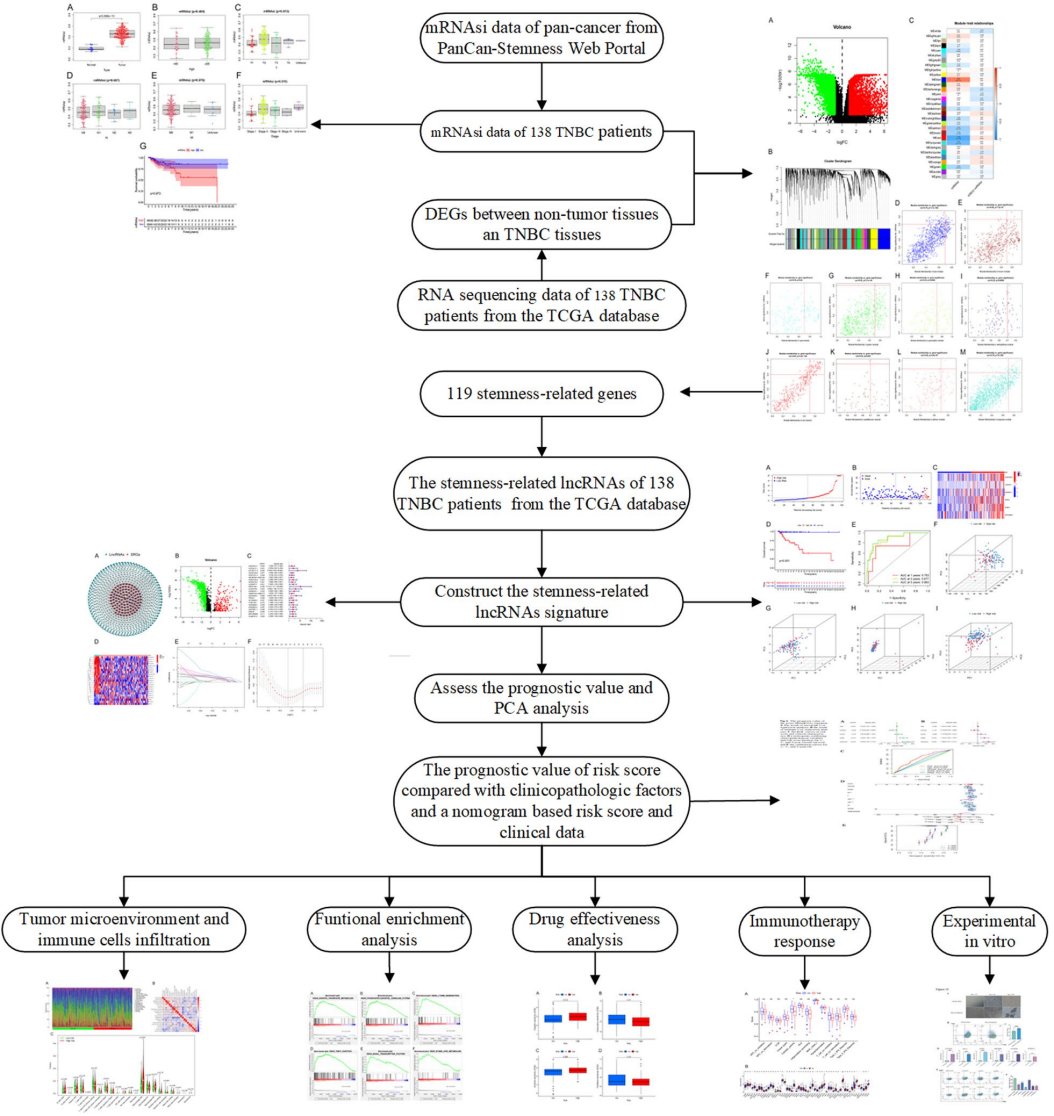
The flow chart of the study

The schematic diagram of this study is illustrated in Fig. 1.

### Data acquisition

RNA sequencing data and corresponding clinical information of BRCA patients were acquired from the TCGA database (https://portal.gdc.cancer.gov) on July 25, 2023. Concurrently, mRNAsi data of BRCA were extracted from PanCanStemness Web portal (https://bioinformaticsfmrp.github.io/PanCanStem_Web) [28]. In addition, we obtained phenotypic information of BRCA TCGA patients from Xena Functional Genomics Explorer (UCSC Xena) (https://xena.ucsc.edu/). Ultimately, we filtered out 138 TNBC patients, with complete clinical data, matching RNA sequencing data and mRNAsi data, for subsequent analysis. Among them, 15 patients had corresponding RNA sequencing and mRNAsi data for non-tumor samples. Additionally, we extracted lncRNA expression profiles of TNBC patients in TCGA based on the lncRNA annotation provided by the GENCODE project [29, 30].

### Differentially expressed genes (DEGs) analysis

To identify differentially expressed genes (DEGs) between non-tumor tissues and TNBC tissues, we employed the R package “limma” with criteria (|logFC|≥ 1.0 and adjusted *P* < 0.05) [31, 32].

### Identification of target modules and SRGs through WGCNA

Target modules were identified through weighted gene co-expression network analysis (WGCNA) using The R package “WGCNA” [33]. WGCNA is a widely used bioinformatics method in genomics and systems biology to identify patterns of gene expression and how these patterns correlate with external traits or conditions, which is extensively used in cancer-related study [13]. Following the filtration of RNA sequence data to mitigate outliers, a similarity matrix was incorporated. Subsequently, a scale-free network was established by determining an optimal soft power value (β = 4). The adjacency matrix was constructed through the following equation: a_ij_ =|S_ij_|^β^ (where a_ij_represents the adjacency matrix between gene i and gene j, S_ij_ denotes the similarity matrix obtained through Pearson correlation of all gene pairs, and β represents the soft power value) [34]. This matrix underwent transformation into a topological overlap matrix (TOM) along with its corresponding dissimilarity (1-TOM). Utilizing the dissimilarity of TOM, we conducted hierarchical clustering with an average linkage approach. The stipulated minimum size for the gene tree diagram was set at 50, consolidating closely related genes into a single module. Additionally, we designated mRNAsi and the epigenetic regulation of mRNAsi as clinical phenotypes. Module eigengene (ME) exhibiting distinct expression patterns were chosen as principal components for modular gene expression principal component analysis (PCA) through feature gene network clustering. Gene significance (GS) was determined to elucidate the association between genes and clinical phenotypes, calculated through the log10 transformation (GS = lgP) of linear regression p-values derived from gene expression and clinical phenotype data. The modular significance (MS) was defined as the mean value of GS. To enhance module capacity, a cut-off (< 0.25) was applied to minimize similarities between modules. Finally, modules of interest, along with the gene membership degree (MM) representing the relationship between a module's own genes and the gene expression profile, were selected. Specific criteria included cor. gene MM > 0.7 and cor. gene |GS|> 0.5, serving as the cut-off values for identifying SRGs within a particular module.

### Identification of SRlncRNAs and differential expression analysis

SRGs were identified, pearson correlation analysis (with a significance threshold of *P* < 0.001 and a correlation coefficient magnitude of |R|> 0.4) was employed to ascertain the correlation between SRlncRNAs and SRGs, Therefore, we obtained SRlncRNAs. Following that, we employed the R package “limma” to identify differentially expressed SRlncRNAs between non-tumor tissues and TNBC tissues, utilizing criteria of |logFC|≥ 1.0 and adjusted *P* < 0.05 [31].

### Construction of the lncRNA-mRNA co-expression network

A lncRNA‒mRNA co-expression network was established utilizing Cytoscape software (version 3.7.2, http://www.cytoscape.org/) [12]. The network was constructed to enable an examination of the relationship between SRGs and SRlncRNAs with Pearson correlation coefficients (with a significance threshold of *P* < 0.001 and |R|> 0.4).

### Construction and validation of the SRlncRNAs prognostic signature

Based on TNBC patients data, we identified prognostic lncRNAs using univariate cox regression analysis (*P* < 0.05). To mitigate overfitting of these SRlncRNAs, we performed least absolute shrinkage and selection operator (LASSO) regression for the selection of significant lncRNAs. Following this, the prognostic model was constructed using multivariate cox regression analysis based on the Akaike Information Criteria (AIC) value after 1000 times cross validation. Six robust lncRNAs were ultimately selected for the construction of the optimal prognostic model. The risk score for TNBC patients was computed using the formula: risk score $={\sum }_{i=1}^{n}\left({\text{expi}}*\mathrm{\beta i}\right)$, where “Exp” denotes the transcriptome expression value of each identified lncRNA, and “β” represents its corresponding coefficient. TNBC patients were stratified into low-risk and high-risk groups based on their median score. The performance of the signature was evaluated using various statistical methods, including Kaplan‒Meier (KM) survival analysis with the log-rank test using the R packages “survminer” and “survival” to assess overall survival (OS) between the two groups. The predictive power of the model was further assessed by the area under the ROC curve (ROC-AUC) using the R package “timeROC” [35]. The distribution of expression patterns among the two groups was examined through Principal Component Analysis (PCA). Additionally, Univariate and multivariate Cox regression analysis were employed to investigate whether the SRlncRNAs signature can serve as an independent prognostic factor in TNBC patients. The predictive accuracy for survival time considering various clinical pathological factors and risk scores was assessed through the receiver operating characteristic (ROC) curve, using the R package “timeROC” [35]. To facilitate clinical decision-making, a nomogram was constructed incorporating risk score and other relevant clinical factors, serving as a quantitative tool for the evaluation of clinical outcomes.

### Gene set enrichment analysis (GSEA)

The potential distinctions in biological processes between high and low-risk groups were investigated through GSEA utilizing the R package “clusterProfiler” with GSEA v4.2.3 software. The gene set annotation C2.cp.kegg.v2023.1.Hs.symbols.gmt was chosen as the reference gene set. A significance threshold of P < 0.05 was considered as statistically significant parameters.

### Assessment of immune infiltration and microenvironment

To explore immune microenvironment in TNBC patients, differences in immune cell infiltration between the two subgroups were evaluated using the CIBERSORT algorithm [36, 37]. The CIBERSORT algorithm is a computational tool designed for characterizing the composition of immune cell populations within complex tissues based on gene expression profiles. The online analysis platform was employed to quantify the relative abundance of 22 immune cell types (including seven T cell types, naive and memory B cells, plasma cells, and NK cells) in TNBC. Simultaneously, we conducted a single-sample Gene Set Enrichment Analysis (ssGSEA) using the R package “gsva” to assess immune-related pathways in both groups [38]. Subsequently, the TIDE algorithm was employed to investigate inhibitors in the immune checkpoint response for TNBC patients (*P* < 0.05) [39].

### Exploration of chemotherapy effect based on the SRlncRNAs signature

Our research focused on improving the operating system in patients with TNBC by employing chemotherapy. Our specific objective was to formulate personalized treatment plans based on risk scores. The half-maximal inhibitory concentration (IC50) characterizes the response of each patient to chemotherapy, as determined using the R package “pRRophetic”, which leverages the Genomics of Drug Sensitivity in Cancer dataset for estimation [40].

### Cell cultures and induction of TNBCSCs

The TNBC cell line (MDA-MB-231) was procured from the Shanghai Cell Bank at the Academy of Sciences (China). The culture conditions of MDA-MB-231 were dulbecco’s modified eagle medium (DMEM; Gibco, Carlsbad, CA, USA), supplemented with 10% fetal bovine serum (FBS) (Gibco) and 1% penicillin–streptomycin (Gibco), in a humidified incubator at 37 °C with 5% CO2. To ensure optimal cell growth and to prevent over-confluency, we conducted our experiments before primary cell line passages reached 40 doublings. Subsequently, enzymatic digestion was employed to convert well-adhered MDA-MB-231 cells in a robust growth state into a single-cell suspension using pancreatin. The supernatant was discarded after centrifugation. The cells were resuspended in a specific medium based on DMEM/F12 (1:1) media (C11330500CP, Gibco), which was supplemented with 0.4% BSA, 20 ng/ml EGF, 10 ng/ml bFGF, and 5 μg/ml recombinant human insulin, then Cells (1*10^5^ per well) are grown in 6-well low adsorption culture plate. The resuspended cells were then cultured in a CO2-regulated incubator at 37 °C for 7–10 days, with the addition of 500 μl specific medium every other day. Throughout this cultivation period, the induction of sphere formation was observed under a microscope, and images were captured. The growth of cells in suspended spherical aggregates were identified as triple-negative breast cancer stem cells (TNBCSCs). Every condition was measured three times. Additional MDA-MB-231 cells (1*10^5 per well) were seeded as a control in a standard 6-well plate and cultured in a complete medium (DMEM supplemented with 10% FBS and 1% penicillin–streptomycin). In the logarithmic growth phase, MDA-MB-231 adherent cells were cultured and conventionally digested at 1000 rpm for 5 min. Following centrifugation, the cells were resuspended in complete culture medium, and the cell density was adjusted to 1 × 10^5 cells/mL, resulting in a single-cell suspension. Subsequently, the cells were seeded into a 6-well plate, with each well receiving 2.5 mL of the single-cell suspension. This process was replicated for each of the three samples. The plate was then placed in a 37 °C incubator with 5% CO2 and left overnight. After the cells adhered to the surface, culture medium was added, and further incubation occurred for an additional 5 days.

### Identification of TNBCSCs

To determine whether TNBCSCs exhibit stem cell-like characteristics, the identification involves observing cell morphology and conducting flow cytometry analysis for the positivity rate of CD44^+^/CD24^−^ cells [41].

### Assessment of the expression of CD44 and CD24 using a flow cytometer

Following the incubation period, cells were collected into EP tubes, appropriately labeled, centrifuged at 1000 rpm for 5 min, with the supernatant being discarded. The cells were washed twice with PBS, centrifuged again at 1000 rpm for 5 min, and resuspended in 500 uL PBS. CD44- FITC and CD24-APC labeling were performed on the resuspended cells. Following a 20 min incubation in the dark at room temperature, 1 mL PBS was added, and the cells were centrifuged at 1000 rpm for 5 min. The supernatant was discarded, and 200 µL PBS was added for resuspension. Subsequently, flow cytometry was employed to detect the expression of CD44 and CD24.

### Quantitative real-time polymerase chain reaction (RT-qPCR)

Total RNA was extracted from cells utilizing MagZol reagent (Magen, Guangzhou, China, cat. No. R4801-01) in accordance with the manufacturer’s instructions. Subsequently, complementary DNA (cDNA) was synthesized through reverse transcription of messenger RNA (mRNA) using the Yeasen Hifair^®^ III 1st Strand cDNA Synthesis SuperMix for qPCR (gDNA digester plus), cat. No.11141ES10. The Quantitative Polymerase Chain Reaction (qPCR) was carried out using 2 × SYBR Green qPCR Master Mix (Low ROX) (Bimake, cat. No. B21702). The standardized conditions for the PCR amplification reactions were as follows: an initial denaturation at 95 ℃ for 10 min, followed by 40 cycles of denaturation at 95 ℃ for 10 s and annealing at 60 ℃ for 30 s. Subsequently, a final extension step was performed at 95 ℃ for 15 s, followed by an annealing step at 60 ℃ for 60 s and a final extension at 95 ℃ for 15 s. The specific primer sequences employed for the target genes, as well as the reference gene (GAPDH), are detailed in Table 1. The relative quantification method (2^−ΔΔCt^) was applied to assess the relative expression of lncRNA.

**Table 1:** Sequences of the primers used in RT-qPCR

LncRNA	Sequence	
GAPDH	Forward primer	CAACGGATTTGGTCGTATTGG
	Reverse primer	TGACGGTGCCATGGAATTT
AC245100.6	Forward primer	CAGTTCTGGAGTTTGGAAGTCTAAGG
	Reverse primer	GATACAGTAGGAAGATGGCAGTCTATG
LINC02511	Forward primer	GCAATGGATGTCGGAGCAGAAG
	Reverse primer	ATGGAAAGGCACTGAAAGGTCTTG
AC092431.1	Forward primer	CAATGGAAGGATGGATGAGGAACC
	Reverse primer	GCAACACACAACCCGTAACATAAC
FRGCA	Forward primer	CCTCCAGTTTCCTCCCACATCC
	Reverse primer	CCACACTCACTCGGACTAGGC
EMSLR	Forward primer	CTCAATGGAAGGACACGGGAAAC
	Reverse primer	GGATCTGTTGCTGGAGAATTACTGG
MIR193BHG	Forward primer	CCCAGCCAGGTTCAGATTTCATAG
	Reverse primer	CACTGCTCTGTTCCTTGCTTCTC

### siRNA

siRNAs were purchased from Sangon Biotech. Transfection of siRNA was carried out according to the manufacturer’s protocol. Briefly, cells in exponential phase of growth were plated in six-well low adsorption tissue culture plates at 1 × 10^5^ cells per well, grown for 96 h, and then transfected with siRNA using lipofectamine RNAimax reagent and DMEM/F12 reduced serum medium. The siRNA sequences were showed in Table 2.

**Table 2:** The siRNA sequences of 6 SRlncRNAs

SiLncRNA		Sequence (5′–3′)	
		Sense	Antisense
SiAC245100.6	1	AGCCUGUUUACGAUAUUGU	ACAAUAUCGUAAACAGGCU
	2	AGGUUUCAAGAUCUAAAGU	ACUUUAGAUCUUGAAACCU
SiLINC02511	1	UGGAACAAUGUCUAACAC	GUGUUAGACAUUGUUCCAG
	2	GGGCUAGAUUUGGUGACUA	UAGUCACCAAAUCUAGCC
SiAC092431.1	1	AUCCGACAUUUUUAAUUUU	AAAAUUAAAAAUGUCGGAU
	2	AAGUUUAAAAUAAAAAUGU	ACAUUUUAUUUUAAACUU
SiFRGCA	1	AAGUUGGAAAAUGAAUGAA	UUCAUUCAUUUUCCAACUU
	2	AUGAAUAUAAAUUAUCAAA	UUUGAUAAUUUAUAUUCAU
SiEMSLR	1	UUGGAAGAAGCAAUUUACA	UGUAAAUUGCUUCUUCCAA
	2	UAGAGAAUUGUGGAAACUG	CAGUUUCCACAAUUCUCUA
SiMIR193BHG	1	GUGCAGAUAUAGACCAUUU	AAAUGGUCUAUAUCUGCAC
	2	UUGAGUAUUAGGCUGAUGU	ACAUCAGCCUAAUACUCAA

### Statistical analysis

All statistical analysis were conducted using R software (version 4.13). The Wilcoxon test was applied to compare the proportional differences in immune-infiltrated cells within the tumor. Pearson correlation analysis was employed to discern relationships between distinct variables. For survival analysis, the Kaplan–Meier (KM) method was utilized. Univariate and multivariate cox regression analysis were performed to examine significant prognostic factors and their independence. The Receiver Operating Characteristic (ROC) curve was employed to evaluate the robustness of the prognostic model for overall survival (OS). Statistical significance was considered when *P* < 0.05, and specific levels of significance were denoted as follows: *P < 0.05, **P < 0.01, ***P < 0.001, and ****P < 0.0001 unless otherwise indicated.

## Results

### mRNAsi and clinical characteristics in TNBC

**Fig. 2:**
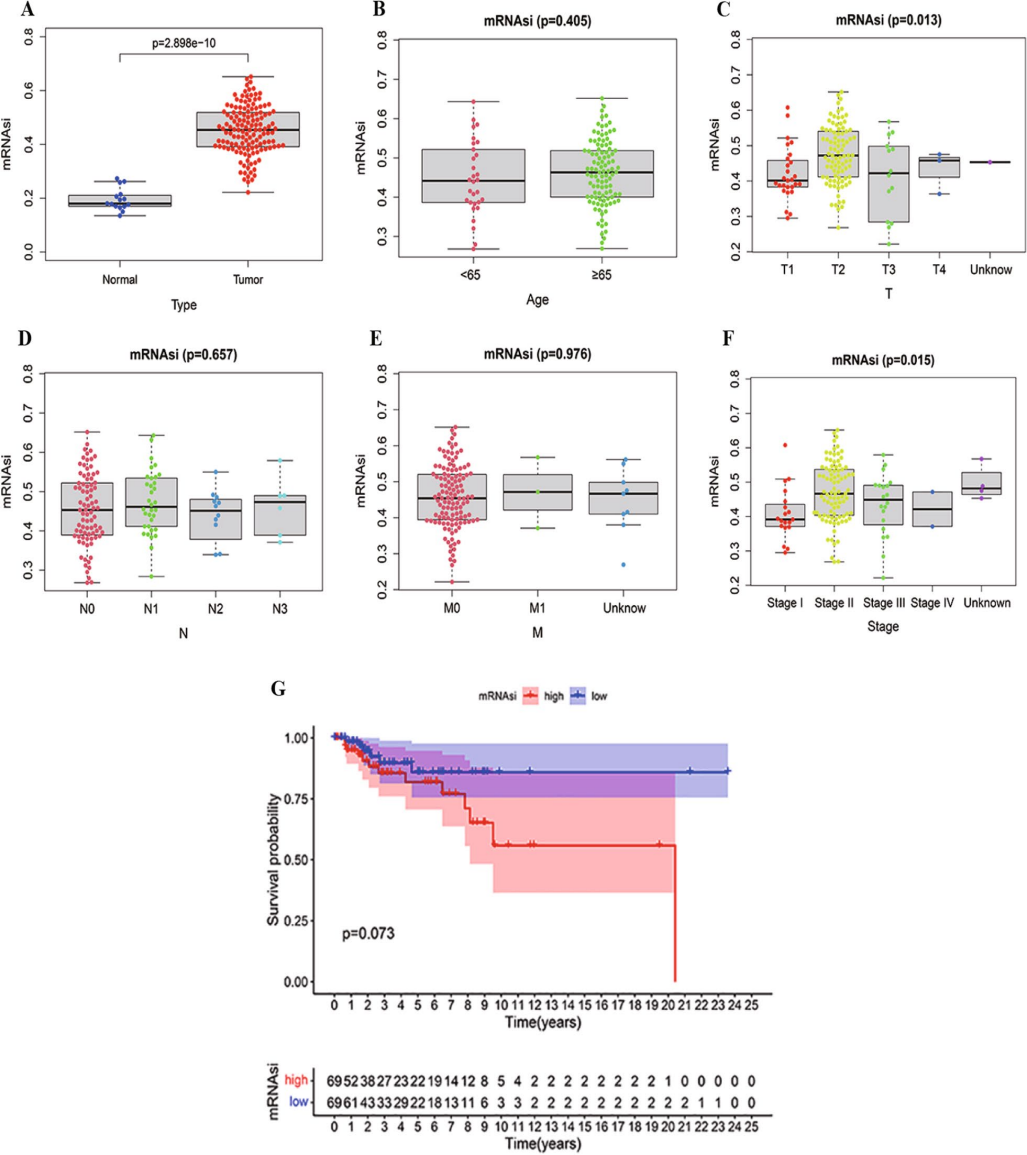
Correlation between mRNAsi and clinical characteristics in TNBC. **A** Differences in mRNAsi between nontumor samples and TNBC tissues. Comparison between mRNAsi expression level and clinical characteristics in HNSCC, including age (**B**), T classification (**C**), N classification (**D**), M classification (**E**) and tumor stage (**F**). **G** Kaplan–Meier curves for TNBC according to mRNAsi. (* p < 0.05, ** *p* < 0.01, *** *p* < 0.001, **** *p* < 0.0001, ns, no significance). *TNBC* triple-negative breast cancer

We acquired mRNAsi data of 138 TNBC patients from the PanCanStemness Web portal. Subsequently, we conducted a comparative analysis of the mRNAsi index between tumor and non-tumor samples, examining various clinicopathological parameters. Our findings revealed a statistically significant elevation in mRNAsi levels in tumor tissues in comparison to normal tissues (Fig. 2A). Moreover, with respect to demographic characteristic, we categorized 138 TNBC patients based on age, gender and tumor stage. Notably, except for T classification and stage, where a statistically significant association was observed (P < 0.001) (Fig. 2C, F), there was no discernible correlation between mRNAsi and age (*P* = 0.405) (Fig. 2B), N classification (*P* = 0.657) (Fig. 2E), or M classification (*P* = 0.976) (Fig. 2E). While mRNAsi determined by Kaplan–Meier (KM) analysis did not exhibit a significant statistical difference (*P* = 0.073 > 0.05), the sufficiently small P-value leaded us to consider that a higher mRNAsi index was associated with poorer overall survival (OS) for TNBC patients (Fig. 2F). The preceding findings suggest that the mRNAsi index may have a discernible impact on the regulation of TNBC.

### Identification of SRGs

**Fig. 3:**
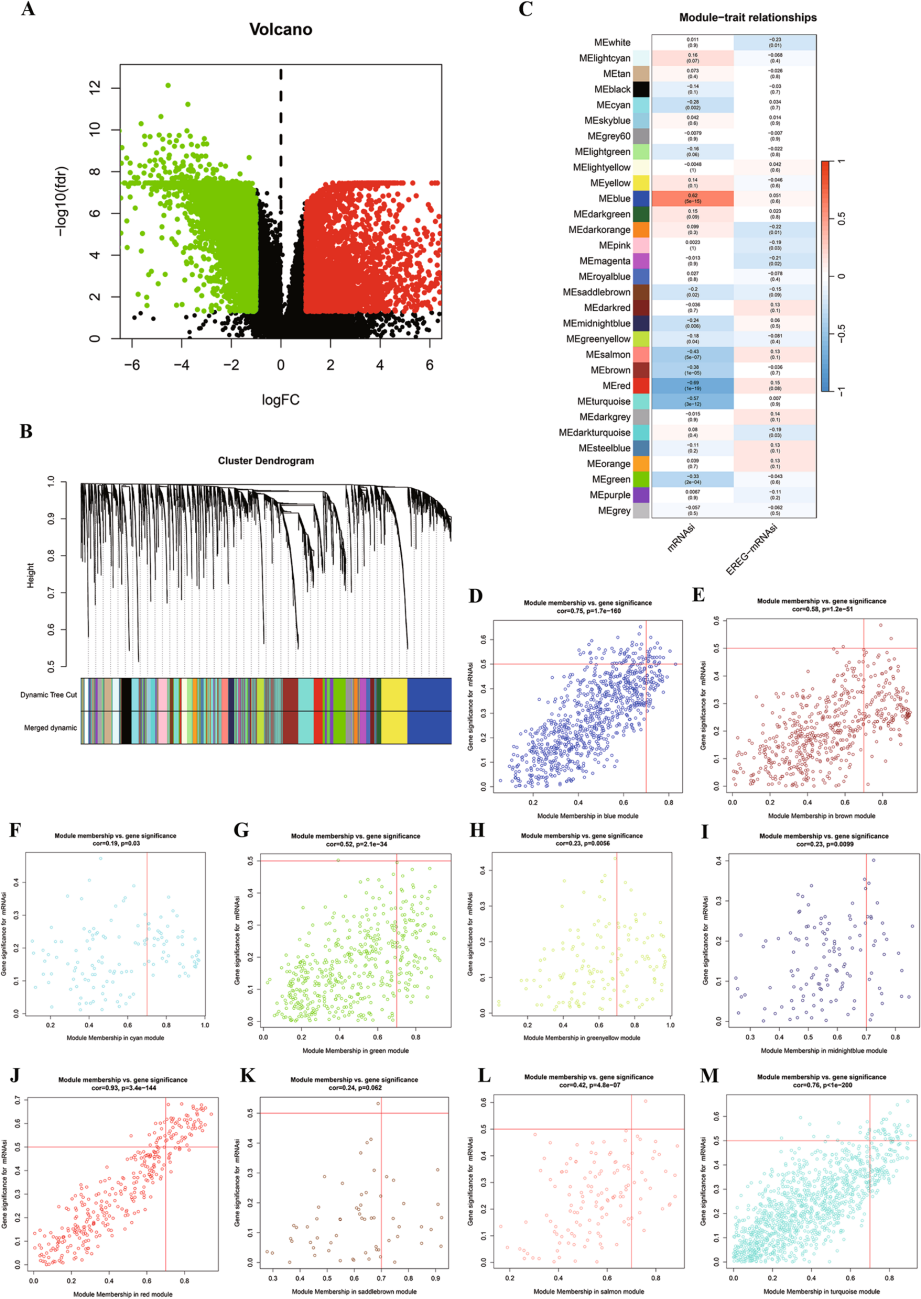
Identification of DEGs and stemness-related key modules in TNBC. **A** the volcano plot of DEGs in the TCGA database. **B** DEGs were clustered into different colors modules. **C** correlation between modules and mRNAsi according to Pearson correlation. **D**–**M** Scatter plot of module eigengenes in the MEcyan, MEbule, MEsaddlebrown, MEmidnightblue, MEgreenyellow, MEsalmon, MEbrow, MEred, MEturquoise and MEgreen modules. *TNBC* triple-negative breast cancer, *DEGs* Differentially expressed genes

Utilizing RNA sequencing data extracted from the TCGA database, we conducted a screening of DEGs between TNBC tissues and non-tumor tissues. Applying a cutoff value (|logFC|≥ 1.0 and adjusted *P* < 0.05), we identified 6629 DEGs, of which 2605 were up-regulated and 4024 were down-regulated genes (Fig. 3A). Subsequently, we employed WGCNA with a soft threshold (β = 4) to establish a scale-free network, allowing the correlation analysis between mRNAsi and the 6629 DEGs in TNBC tissues and non-tumor tissues of the TCGA database. This analysis revealed 32 modules for further investigation (Fig. 3B). Figure 3C illustrated the statistical methodology employed for identifying modules closely associated with the mRNAsi index. WGCNA results depicted the correlation level (R^2^ value) between TNBC gene expression and the mRNAsi index in each module. Consequently, we selected ten modules (MEcyan, MEbule, MEsaddlebrown, MEmidnightblue, MEgreenyellow, MEsalmon, MEbrow, MEred, MEturquoise and MEgreen modules) (*P* < 0.05) as the primary focus for subsequent studies (Fig. 3D–M). Finally, applying a threshold of |GS|> 0.5 and MM > 0.7, we identified 119 SRGs in these modules.

### Identification of prognostic SRlncRNAs

**Fig. 4:**
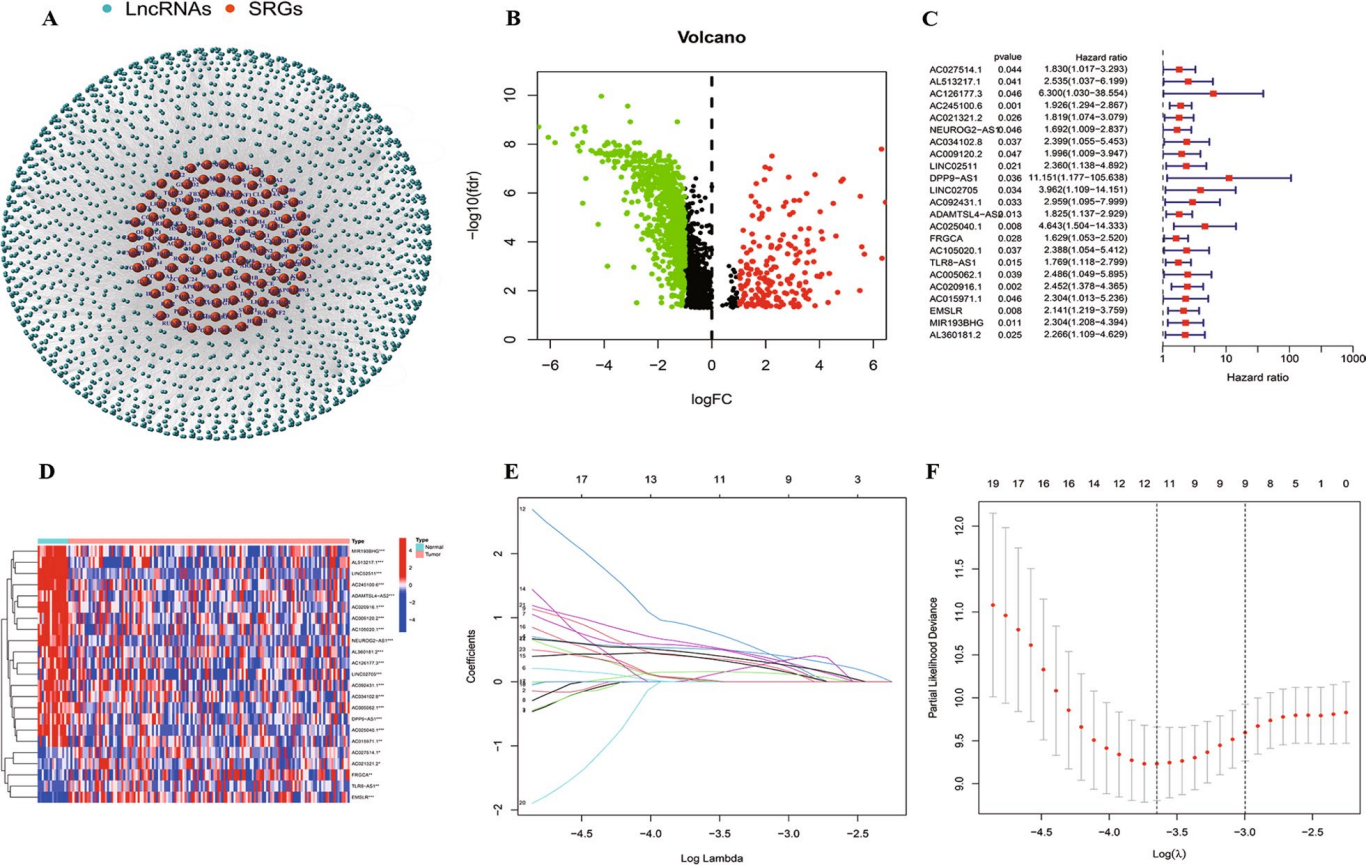
Identification of stemness-related lncRNAs signature in TNBC. **A** The network of SRGs and SRlncRNAs. **B** the volcano plot of SRlncRNAs. **C** The forest plot of prognostic-related SRlncRNAs. **D** Heatmap of prognostic-related SRlncRNAs. **E** LASSO coefficient profiles of SRlncRNAs. **F** The partial likelihood deviance with changing of log(λ). (* p < 0.05, ** *p* < 0.01, *** *p* < 0.001, **** *p* < 0.0001, ns, no significance). *TNBC* triple-negative breast cancer, *SRGs* stemness-related genes, *SRlncRNAs* stemness-related lncRNAs

Utilizing clinicopathological data, RNA sequencing data, retrieved from the TCGA database for 138 TNBC patients, we identified a total of 16,876 lncRNAs and 19,938 mRNAs. Conducting Pearson correlation analysis between lncRNAs and 119 SRGs (*P* < 0.001 and |R|> 0.4), we derived 1982 SRlncRNAs. Subsequently, we established a co-expression network illustrating the relationships between 119 SRGs and 1982 SRlncRNAs (Fig. 4A). We performed differential analysis on these obtained SRlncRNAs, and We obtained 922 differentially expressed SRlncRNAs, which are considered potential prognostic SRlncRNAs.

### Construction and validation of the SRlncRNAs signature

Based on the results of univariate Cox regression analysis, 23 SRlncRNAs were identified as being associated with overall survival (OS), as depicted in the forest plot and heatmap (Fig. 4C, D). All of these 23 SRlncRNAs were deemed closely linked to the unfavorable prognosis of TNBC patients based on a Hazard Ratio (HR) greater than 1. In order to mitigate multicollinearity, lasso regression analysis was employed to analyze 23 SRlncRNAs, during 1000 times cross validation, the least AIC score facilitated the identification of the optimal lncRNAs signature, we ultimately identified six SRlncRNAs (AC245100.6, LINC02511, AC092431.1, FRGCA, EMSLR and MIR193BHG) to establish the prognostic model according to the AIC value (AIC = 127.91). Figure 4E, F presented the cvfit and lambda curves, respectively. Subsequently, the risk score of TNBC patients was computed utilizing the following formula: Risk score = AC245100.6 * 0.58910 + LINC02511 * 0.81093 + AC092431.1 * 1.83066 + FRGCA * 0.67583 + EMSLR * 1.07227 + MIR193BHG * 0.68825.

**Fig. 5:**
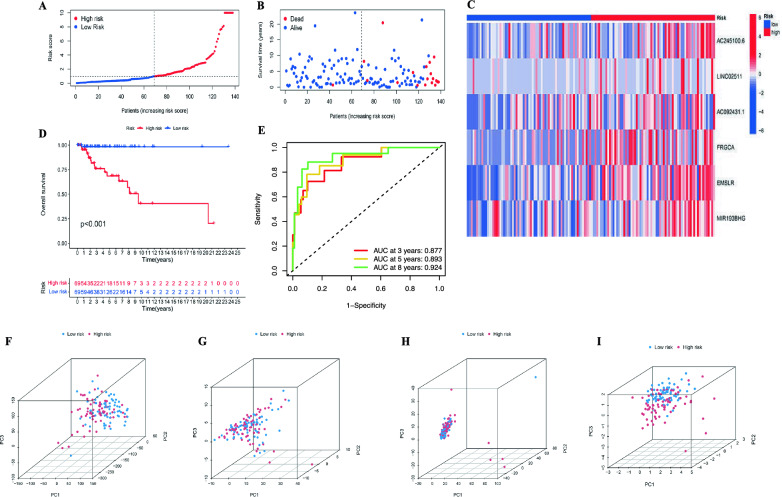
A risk model for outcome prediction. **A** The distribution of the risk scores for each patient. **B** the distributions of the overall survival status for every patient. **C** The heatmap of 6 SRlncRNAs expression. **D** Kaplan–Meier curves for the overall survival of patients in the high- and low-risk groups. **E** Accuracy of the risk signature in predicting 3-, 5-, and 8-year ROC curves. **F** PCA of all examined genes expression. **G** PCA of all SRGs expression. **H** PCA of SRlncRNAs expression. **I** PCA of the prognostic 6 SRlncRNAs signature. *TNBC* triple-negative breast cancer, *SRGs* stemness-related genes, *SRlncRNAs* stemness-related lncRNAs, *PCA* Principal components analysis

Next, the prognostic accuracy of the model was evaluated. The risk scores for each individual in the TNBC cohort were computed using a designated formula and organized in ascending order. Subsequently, individuals in the TNBC cohort were categorically assigned to two distinct groups, namely the high-risk and low-risk groups, based on whether their scores were above or below the median values. The survival outcomes, risk status and lncRNA expression levels of all patients were illustrated in Fig. 5A–C. The outcomes derived from the Kaplan–Meier (KM) survival analysis revealed that TNBC patients exhibited a diminished survival duration in the high-risk group (Fig. 5D), which suggested that TNBC patients experienced a more favorable prognosis in the low-risk group. An ROC curve was constructed to compute AUC for the risk score. The findings indicated that the AUC values for the risk score at 3, 5, and 8 years were 0.877, 0.893, and 0.924, respectively (Fig. 5E). PCA was employed to discern distinctions between the two subgroups, utilizing all genes, 119 SRGs, 1982 SRlncRNAs, and the 6-SRlncRNAs signature (Fig. 5F–I). The PCA outcomes revealed that, particularly in the analysis of the 6-SRlncRNAs signature (Fig. 5I), both the low and high-risk groups exhibited more pronounced divergence in distinct directions compared to the other analysis (Fig. 5F–H). These findings suggested that the risk model effectively stratified TNBC patients into two groups (low and high-risk), demonstrating a complete separation in their stemness status.

### Construction and verification of a nomogram in TNBC patients

**Fig. 6:**
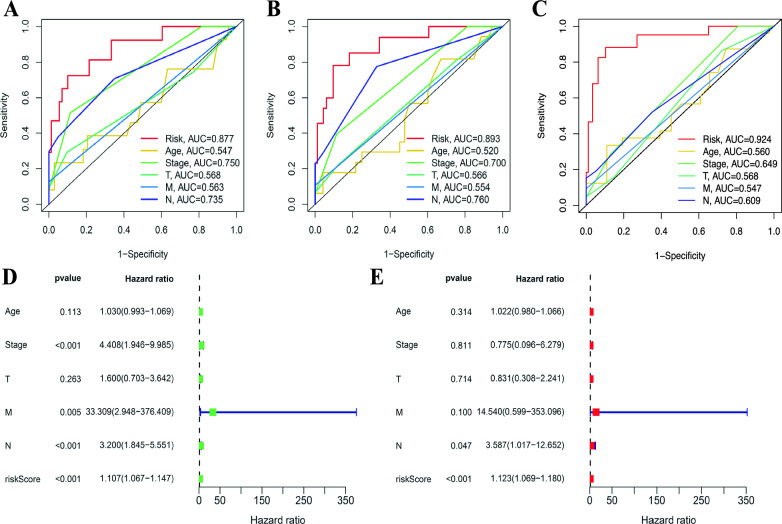
The prognosis value of the novel SRlncRNAs signature. **A** The ROC curves of risk score and clinical characteristics at 3-year. **B** The ROC curves of risk score and clinical characteristics at 5-year. **C** The ROC curves of risk score and clinical characteristics at 8-year. **D** The result of univariate Cox regression analysis. **E** The result of multiple Cox regression analysis. *SRlncRNAs* stemness-related lncRNAs

**Fig. 7:**
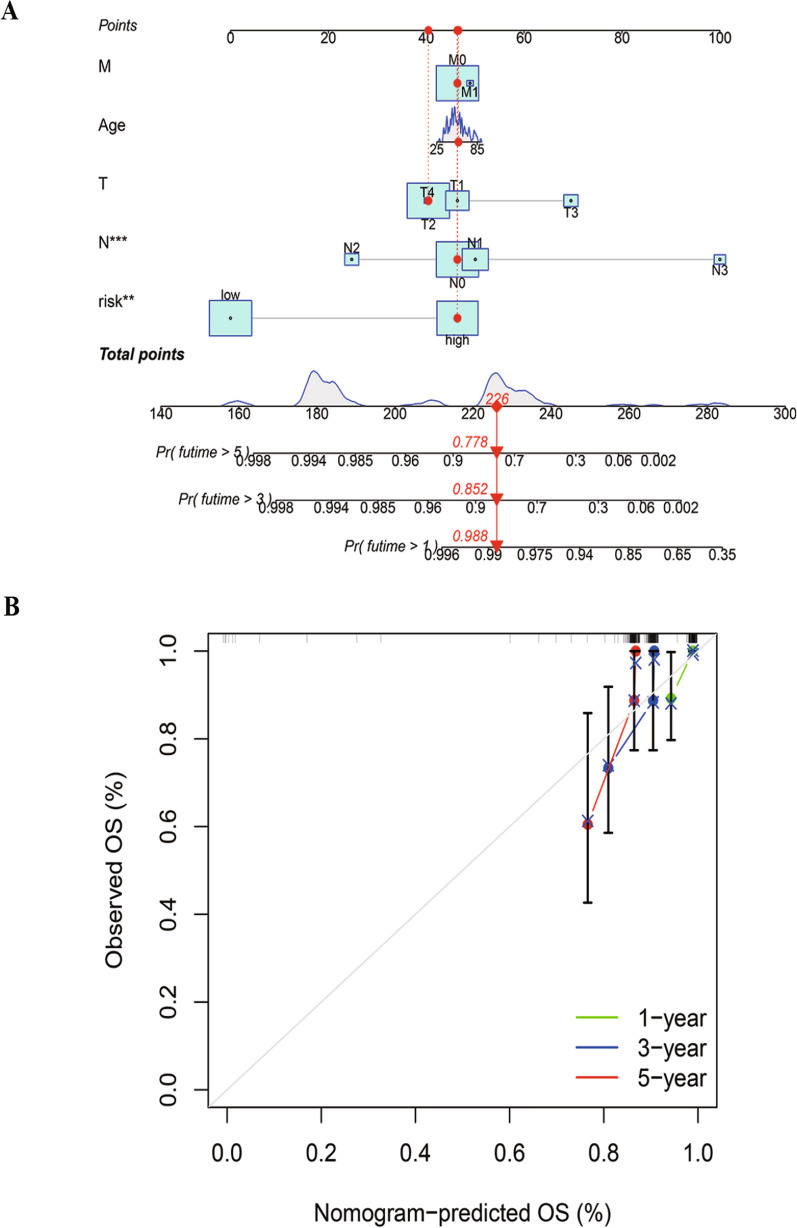
Construction and verification of the nomogram in TNBC. **A** A nomogram combining clinicopathological variables and risk score predicts the 1-, 3-, and 5- year overall survival. **B** The calibration curves for 1‐, 3‐, and 5‐year OS. (* p < 0.05, ** *p* < 0.01, *** *p* < 0.001, **** *p* < 0.0001, ns, no significance). *TNBC* triple-negative breast cancer

The predictive specificity and sensitivity of risk scores for TNBC patients at 3, 5, and 8 years were subsequently assessed using the AUC. The results indicated that the AUC values for the risk scores all surpassed those of other clinicopathological factors (the AUC of 3 year: 0.877; the AUC of 5 year: AUC = 0.893; the AUC of 8 year: 0.924) (Fig. 6A–C). To ascertain whether the risk score can be considered an independent prognostic factor influencing the outcomes of TNBC patients, we incorporated clinical factors into both univariate and multivariate cox regression analysis. Univariate cox regression analysis revealed that stage (HR, 4.408; CI 1.946–9.985; *P* < 0.001), M (HR, 33.309; CI 2.948–376.409; *P* = 0.005), N (HR, 3.200; CI 1.845–5.551; *P* < 0.001) and risk score (HR, 1.107; CI 1.067–1.147; *P* < 0.001) exhibited significant associations with overall survival (OS) (Fig. 6D). Multivariate cox regression analysis demonstrated that N (HR, 3.587; CI 1.069–12.652; *P* = 0.047) and risk score (HR, 1.123; CI 1.069–1.180; *P* < 0.001) independently predicted OS in TNBC patients (Fig. 6E). subsequently, a prognostic nomogram was executed to evaluate the prognostic outcomes of patients diagnosed with TNBC at 1, 3, and 5 years post-diagnosis in the clinical setting (Fig. 7A). Following this, the calibration curves demonstrated satisfactory calibration (Fig. 7B).

### Gene set enrichment analysis (GSEA)

**Fig. 8:**
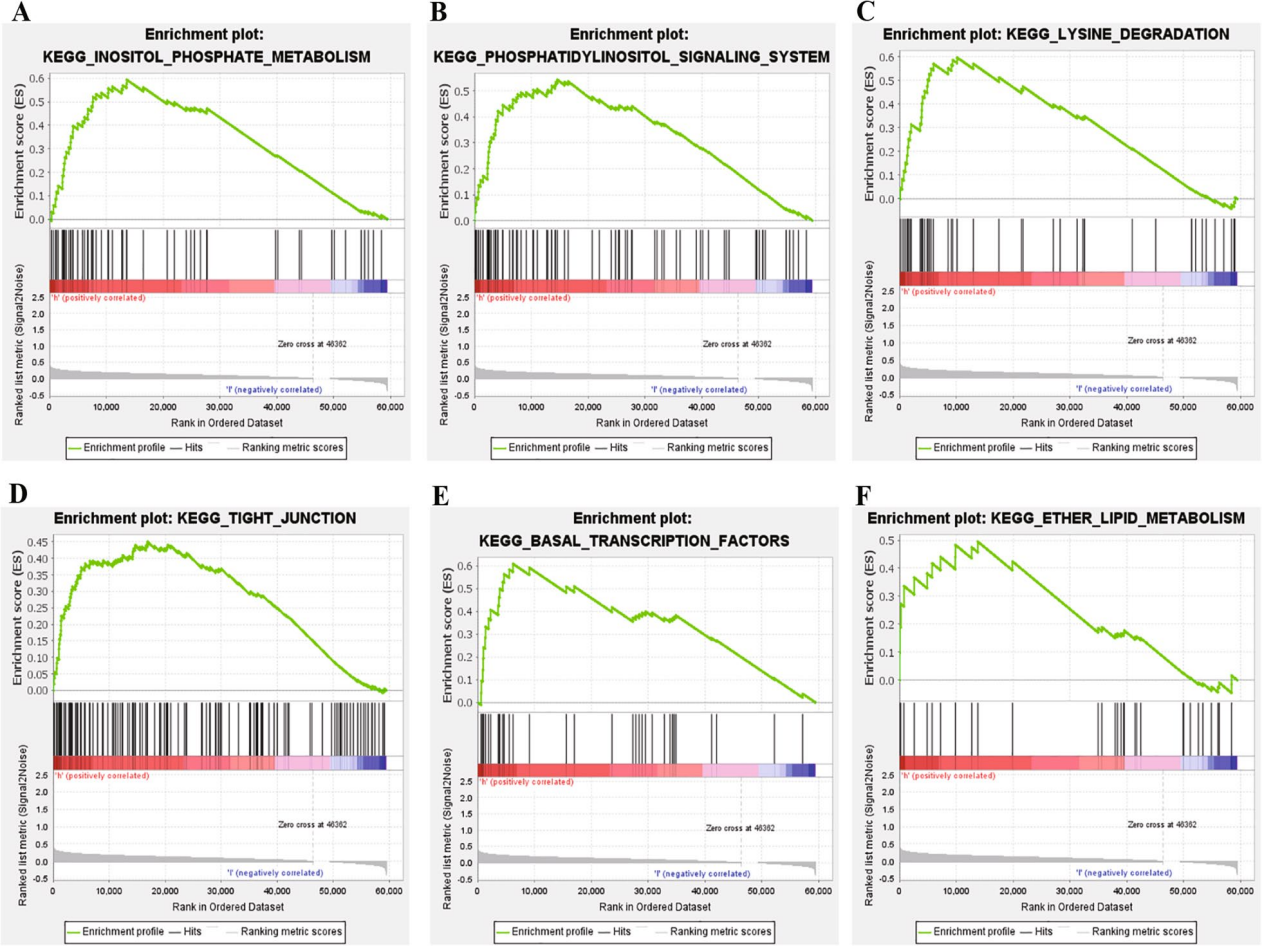
Gene set enrichment analysis (GSEA) of the high- and low-risk groups based on the prognostic model. **A**–**F** The significantly enriched KEGG pathways in the high-risk group

The variation in signaling pathways between the low-risk and high-risk groups was scrutinized through GSEA. The GSEA results unveiled that in the high-risk group, among 178 signaling pathways, a total of 158 exhibited upregulation. Notably, six signaling pathways, including inositol phosphate metabolism, phosphatidylinositol signaling system, lysine degradation, tight junction, basal transcription factors and ether lipid metabolism, demonstrated significant enrichment at a nominal significance threshold (*P* < 0.05) (Fig. 8). In contrast, in the low-risk group, among the same 178 signaling pathways, only 20 displayed upregulation, and no pathways demonstrated significant enrichment at a similar nominal significance level (*P* < 0.05).

### The immune cells infiltration landscape in TNBC patients

**Fig. 9:**
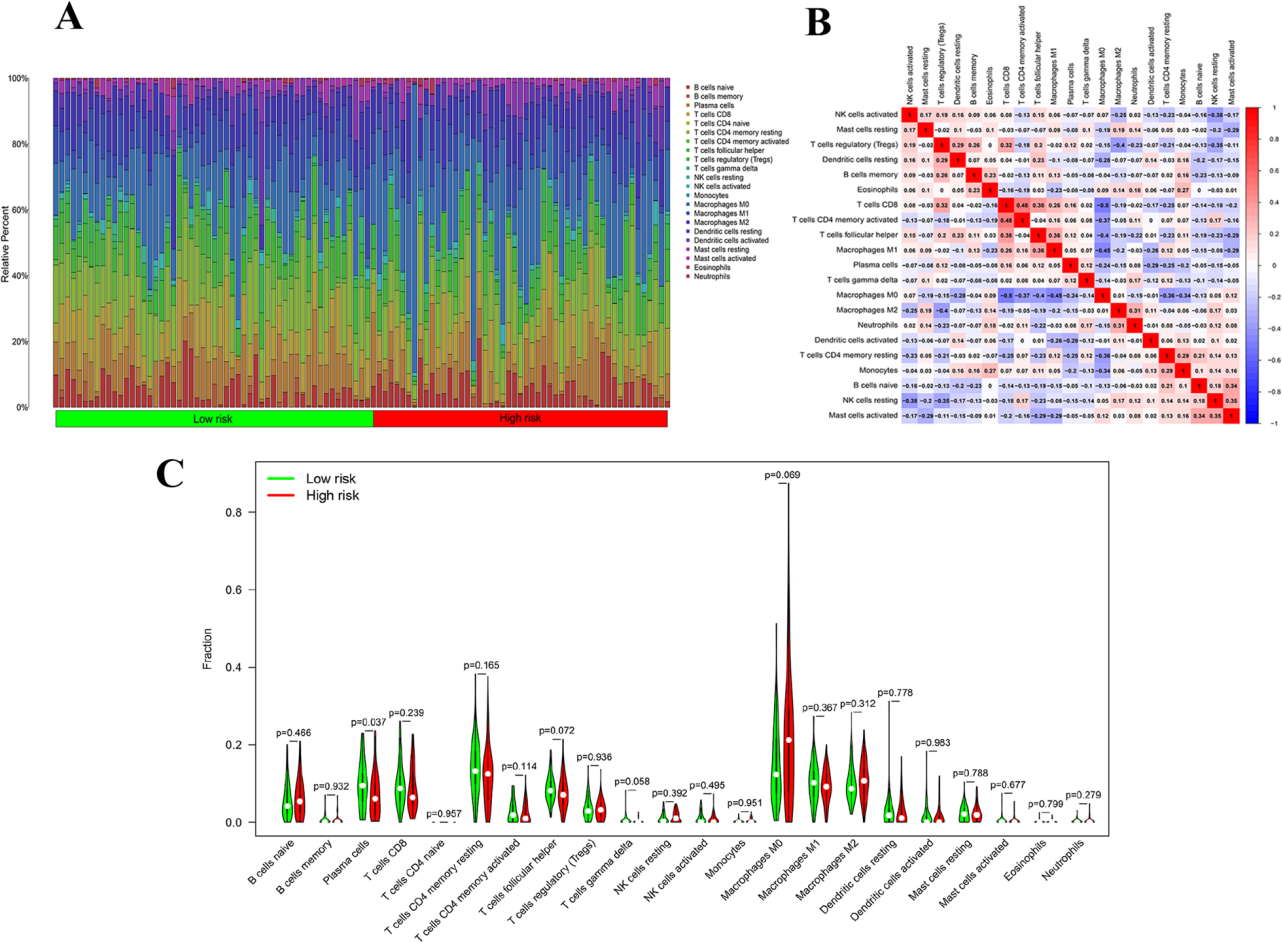
Analysis of the immune cell infiltration landscape in TNBC patients calculated by CIBERSORT. **A** The proportions of 22 tumor infiltrating immune cells in individual TNBC patients. **B** Correlations among immune cells in TNBC patients. **C** Violin diagram showing the immune cell composition of different groups. (* *p* < 0.05, ** *p* < 0.01, *** *p* < 0.001, **** *p* < 0.0001, ns, no significance). *TNBC* triple-negative breast cancer

Immune cells play a crucial role in both tumor formation and prognosis. In our exploration of immune cell infiltration patterns in TNBC patients in the TCGA cohort, we analyzed the data derived from the TCGA database. Our assessment revealed substantial variations in the distribution of 22 distinct immune cell types in the tumor tissues of TNBC patients (Fig. 9A). Furthermore, we conducted an analysis to elucidate the correlations between infiltrating immune cells in TNBC patients. Notably, activated natural killer (NK) cells exhibited positive correlations with resting mast cells, regulatory T cells (Tregs), resting dendritic cells, memory B cells, eosinophils, CD8 + T cells, T follicular helper cells, M0 macrophages, and neutrophils (Fig. 9B). Upon comparing immune cell proportions across different risk groups, we observed that the low-risk group displayed elevated levels of plasma cells (Fig. 9C).

**Fig. 10:**
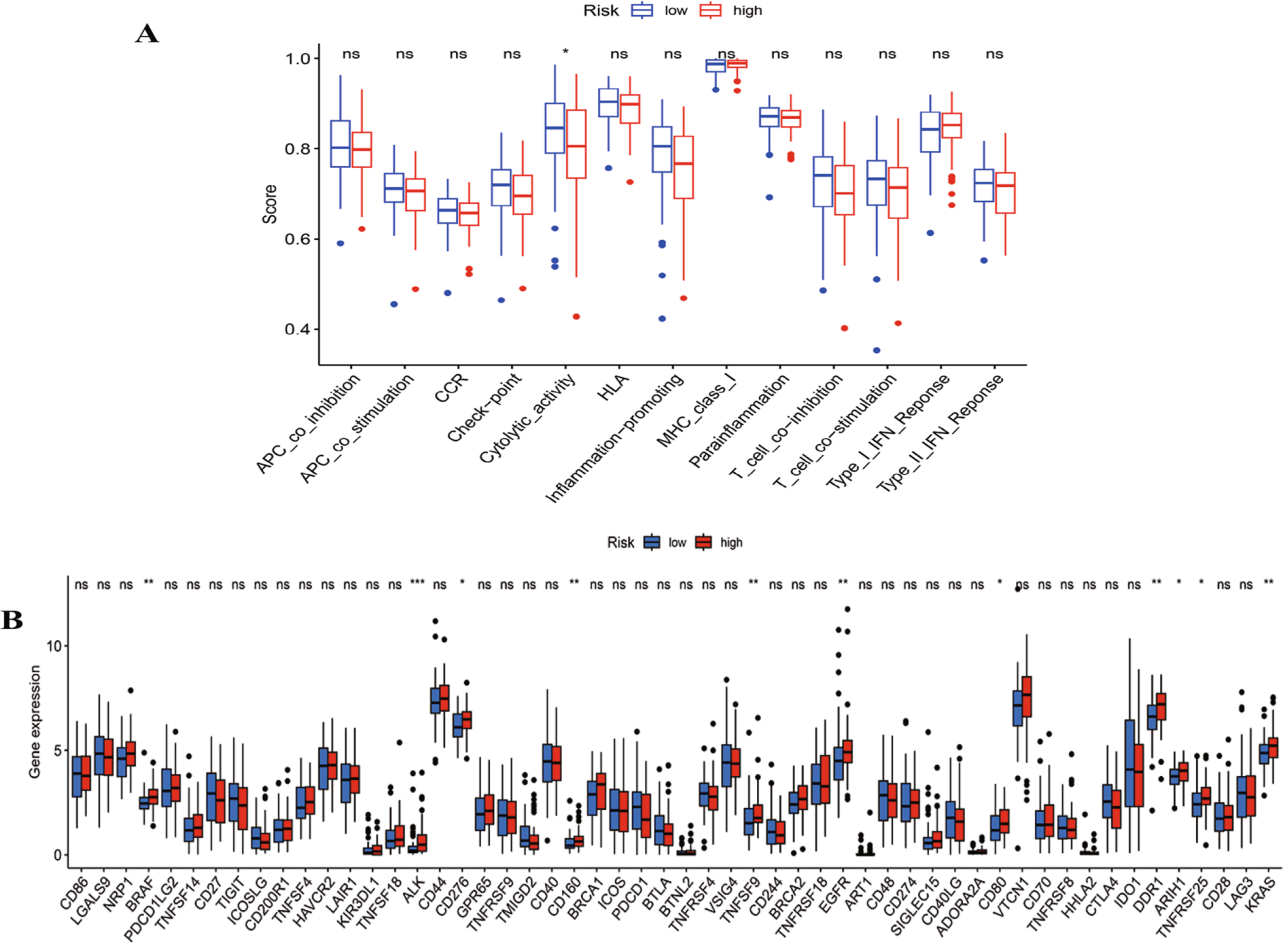
Immunity correlation analysis of the risk score in TNBC patients based on the ssGSEA scores. **A** Immune functional differences between the low-risk and high-risk groups. **B** The difference of common immune checkpoints expression in the two groups. (* *p* < 0.05, ** *p* < 0.01, *** *p* < 0.001, **** *p* < 0.0001, ns, no significance). *TNBC* triple-negative breast cancer

Next, we assessed variations in the enrichment levels of 13 pathways related to immune function between the aforementioned groups. Notably, a predominant focus was observed in cytolytic activity, which contributes to inflammation (Fig. 10A). Additionally, we examined alterations in the expression of common immune checkpoint genes within these groups. The results indicated significant differences in 11 immune checkpoint genes (BRAF, ALK, CD276, CD160, TNFSF9, EGFR, CD80, DDR1, ARIH1, TNFSF25, and KRAS) between the two groups (Fig. 10B).

### Sensitivity assessment of common chemotherapeutic drugs

**Fig. 11:**
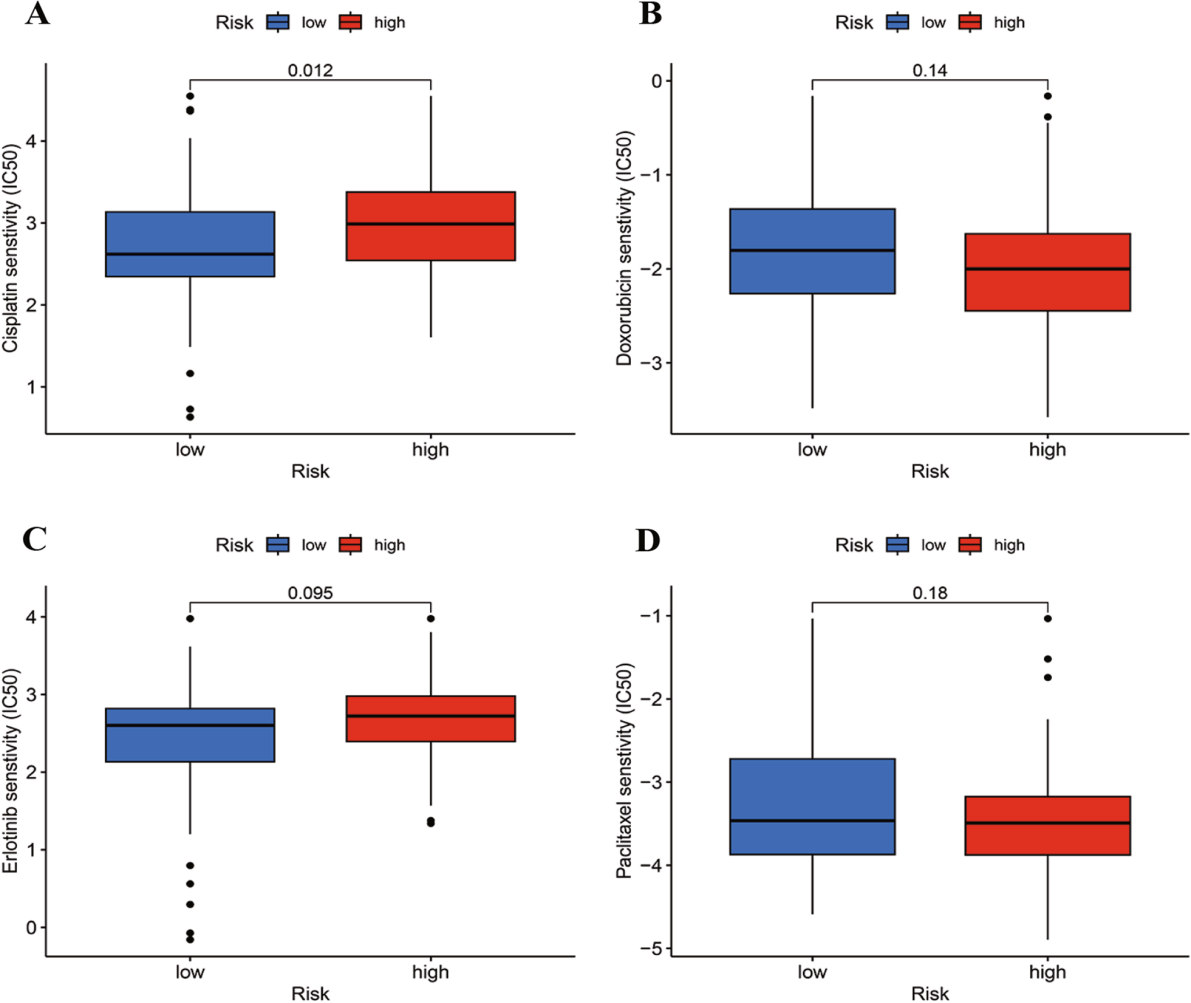
The chemotherapeutic responses of the two groups to four common anticancer drugs. **A** Cisplatin. **B** Docetaxel. **C** Eriotinib. **D** Paclitaxel

It is widely acknowledged that cisplatin, docetaxel, paclitaxel, and erlotinib are among the most commonly prescribed chemotherapy drugs for TNBC patients [42]. Consequently, through an analysis of the correlation between the risk score and the efficacy of these extensively recognized anticancer medications, it was observed that individuals in the low-risk group exhibited heightened sensitivity to cisplatin (Fig. 11). This finding held potential significance for tailoring personalized therapeutic approaches for TNBC patients.

### Identification of induced stemness in TNBC CSCs

**Fig. 12:**
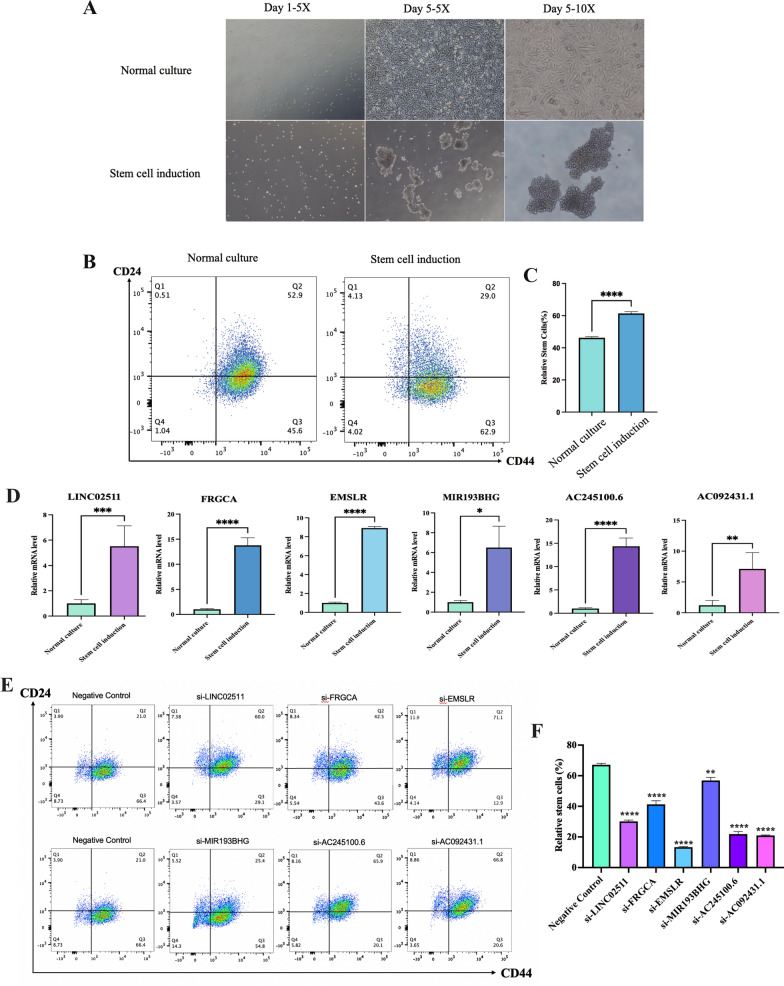
Identification of TNBCSCs and the qRT-PCR results of these SRlncRNAs relative expressing levels in in vitro. **A** The morphological changes of MDA-MB-231 cells following induction of stemness. **B** Flow cytometry results about the proportion of CD44 + CD24- cells between the control group and TNBCSCs. **C** The statistical analysis results of flow cytometry outcomes between the control group and the TNBCSCs group (**D**) the qRT-PCR results of these SRlncRNAs in TNBCSCs. **E** Flow cytometry results about the proportion of CD44 + CD24- cells between the TNBCSCs control group and the TNBCSCs group respectively silenced target lncRNA. **F** The statistical analysis results of flow cytometry outcomes between the TNBCSCs control group and the TNBCSCs group respectively silenced target lncRNA. (* *p* < 0.05, ** *p* < 0.01, *** *p* < 0.001, **** *p* < 0.0001, ns, no significance). *TNBCSCs* triple-negative breast cancer stem cells

The findings from inverted phase-contrast microscopy revealed that initially induced MDA-MB-231 cells by stem cells displayed a single, rounded cell state suspended within the culture medium. After 1–2 days, several cells gradually aggregated into loosely clustered structures resembling bead-like formations. By day 5, these structures gradually transformed into more regularly shaped grape-like cell clusters, with clearer cell structures and strong translucency. Between 5 and 10 days, cells proliferated rapidly, and the cell clusters progressively enlarged. The boundaries between cells became blurred or covered by newly proliferated cells, forming a more densely organized, round or elliptical three-dimensional structure with poor translucency (Fig. 12A). The flow cytometry results demonstrated that, following serum-free suspension culture, stem cell marker in ovarian cancer cells was induced. The proportion of CD44^+^CD24^−^ cells in MDA-MB-231 cells was (46.23 ± 0.34) %, whereas the proportion of CD44^+^CD24^−^ cells in TNBC stem cells was (61.33 ± 1.80) %. The difference was statistically significant (*P* < 0.05) (Fig. 12B).

### Investigation of in vitro alterations in 6 SRlncRNAs

Expression levels of 6 SRlncRNAs were separately examined in MDA-MB-231 cells and MDA-MB-231 stem cells using RT-qPCR. The results revealed a significant increase in the expression of AC245100.6, LINC02511, AC092431.1, FRGCA, EMSLR and MIR193BHG in the MDA-MB-231 stem cell group compared to the MDA-MB-231 (*P* < 0.05) (Fig. 12C). In addition, when silent six SRlncRNAs, we observed a statistically significant decrease in relative MDA-MB-231 stem cell numbers (*P* < 0.05) (Fig. 12E, F).

## Discussion

TNBC represents an exceptionally aggressive subtype distinguished by the lack of expression of the ER, PR and HER2 [43]. The molecular heterogeneity of TNBC poses challenges in treatment strategies. Tumor heterogeneity is partly derived from the presence and activity of CSCs [44]. In the realm of tumorigenesis, metastasis, and post-treatment relapse, CSCs, constituting a minority subset within the tumor cell population, emerge as pivotal contributors, and abundant evidence attests to the paramount role played by CSCs in instigating chemoresistance to chemotherapy and the subsequent resurgence in various types of cancer, including breast cancer [44–47]. The malignant characteristics of tumors are functionally associated with the expression of stem cell markers and CSCs-specific transcriptional patterns, and these molecular signatures exhibit a high prognostic value for overall patient survival [48]. Numerous reports have documented the use of SRGs as prognostic markers [49]. Nevertheless, a notable research gap exists in the exploration of lncRNAs based on stemness in TNBC. The identification of novel biomarkers and the pursuit of more efficacious therapeutic targets in TNBC remain imperative and warranted.

We explored the clinical significance of mRNAsi in predicting the prognosis of TNBC. Our investigation revealed a significantly elevated mRNAsi in TNBC tissues. Furthermore, Kaplan–Meier survival analysis demonstrated that lower mRNAsi was associated with a more favorable survival prognosis. Our findings aligned with previously reported results in the literature [50]. These findings implied that stemness played a crucial role in TNBC.

Utilizing paired clinical data, corresponding RNA-Seq data, and mRNA-based stemness index (mRNAsi) from 138 TNBC patients, we identified 119 SRGs. Following a comprehensive analysis, we further pinpointed 6 resilient predictive lncRNAs (AC245100.6, LINC02511, AC092431.1, FRGCA, EMSLR, and MIR193BHG) to formulate a prognostic model, and the model effectively stratified TNBC patients into low-risk and high-risk groups. The high-risk group exhibited poorer overall survival, indicating the potential of SRlncRNAs as prognostic markers. The signature’s robustness was validated through various statistical methods, including Kaplan–Meier survival analysis and receiver operating characteristic (ROC) curve analysis.

Some reasons might be attributed to distinct underlying mechanisms among various subtypes of risk scores. In our quest to unravel potential mechanisms, we conducted GSEA, the result revealed the activation of numerous signaling pathways associated with tumorigenesis, including inositol phosphate metabolism, phosphatidylinositol signaling system, lysine degradation, tight junction, basal transcription factors and ether lipid metabolism were enriched in the high-risk group. Moreover, to explored the immune microenvironment in TNBC, we assessed the variations in the distribution of 22 distinct immune cell types between the two groups, the result showed plasma cells was notably higher in the low-risk group compared to the high-risk group. Common knowledge dictated that the ability of plasma cells to generate specific antibodies provided the immune system with a targeted defense mechanism against a wide range of antigens and plasma cells played a vital role in maintaining immune system function and overall health [51]. Those phenomenons listed above may help to explore the disparity in prognosis between the two groups. And these findings also suggested that SRlncRNAs might influence critical cellular processes contributing to TNBC progression.

In order to investigate potential novel personalized therapeutic approaches for TNBC, we assessed the expression of immune checkpoint genes and identified differences between the two risk groups. We found immune checkpoint genes (BRAF, ALK, CD276, CD160, TNFSF9, EGFR, CD80, DDR1, ARIH1, TNFSF25, and KRAS) were up-regulated in the high-risk group, which indicated that treatment with anti- immune checkpoint genes therapy may be beneficial for TNBC patients. Furthermore, various computational methods have been applied to drug screening for multiple diseases [52–55], our research investigated the sensitivity of TNBC patients to common chemotherapeutic drugs. The low-risk group showed heightened sensitivity to cisplatin, suggesting that the SRlncRNA signature could aid in personalized treatment planning for TNBC patients.

In vitro experiments induced stemness in TNBC stem cells, confirming the stem cell-like characteristics of the identified SRlncRNAs. The expression of these SRlncRNAs was significantly increased in TNBC stem cells compared to non-stem TNBC cells, while the proportion of stem cells in TNBC stem cells with silenced SRlncRNAs decreased, suggesting that these lncRNAs might play key roles in TNBC. At present, there are no relevant reports on the biological research of lncRNA AC245100.6, lncRNA LINC02511 and lncRNA AC092431.1. Liao et al., reported that lncRNA FRGCA was highly expressed in colon adenocarcinoma and correlated with poor prognosis [56]. Hegre, S et al., reported that an oncogenic role of EMSLR, possibly by interfering with the progression from G1 to the S phase of the cell cycle [57]. Zhou et al., reported that MIR193BHG was one of the six pyroptosis-related lncRNAs prognostic signature for renal clear cell cancer prognosis prediction [58]. However, the roles of these six SRlncRNAs had not been reported in TNBC, the specific stem cell-like characteristics of 6 lncRNAs in TNBC merit further investigation, paving the way for future exploration in our research endeavors.

However, our study has several limitations. Firstly, reliance on the TCGA database for our analysis, despite a substantial sample of TNBC patients, would benefit from additional external validation cohorts for more robust analyses. Finally, the biological mechanisms of SRlncRNAs remain unclear, as no cell or animal experiments have been conducted; therefore, future studies will include in vitro and in vivo investigations to elucidate these mechanisms.

## Conclusion

Our study provided insights into the potential roles of SRlncRNAs in TNBC, offering a novel prognostic signature and highlighting their association with immune responses and chemotherapy sensitivity. Further validation and functional studies are warranted to fully elucidate the mechanisms underlying SRlncRNAs in TNBC, paving the way for personalized therapeutic interventions.

## Data Availability

The article and supplemental material encompass the dataset employed in our research. The original data is accessible at https://portal.gdc.cancer.gov. For any further inquiries, kindly reach out to the corresponding author directly.

## References

[1] Sung H, Ferlay J, Siegel RL, Laversanne M, Soerjomataram I, Jemal A, Bray F (2021). Global cancer statistics 2020: GLOBOCAN estimates of incidence and mortality worldwide for 36 cancers in 185 countries. CA Cancer J Clin.

[2] Wang F, Xiang Z, Huang T, Zhang M, Zhou WB (2020). ANLN directly interacts with RhoA to promote doxorubicin resistance in breast cancer cells. Cancer Manage Res.

[3] Wang Z, Zhang M, Shan R, Wang YJ, Chen J, Huang J, Sun LQ, Zhou WB (2019). MTMR3 is upregulated in patients with breast cancer and regulates proliferation, cell cycle progression and autophagy in breast cancer cells. Oncol Rep.

[4] Zhang M, Xiang Z, Wang F, Shan R, Li L, Chen J, Liu BA, Huang J, Sun LQ, Zhou WB (2020). STARD4 promotes breast cancer cell malignancy. Oncol Rep.

[5] Derakhshan F, Reis-Filho JS (2022). Pathogenesis of triple-negative breast cancer. Annu Rev Pathol.

[6] Sun X, Tang H, Chen Y, Chen Z, Hu Z, Cui Z, Tao Y, Yuan J, Fu Y, Zhuang Z, He Q, Li Q, Xu X, Wan X, Jiang Y, Mao Z (2023). Loss of the receptors ER, PR and HER2 promotes USP15-dependent stabilization of PARP1 in triple-negative breast cancer. Nat Cancer.

[7] Bassiouni R, Idowu MO, Gibbs LD, Robila V, Grizzard PJ, Webb MG, Song J, Noriega A, Craig DW, Carpten JD (2023). Spatial transcriptomic analysis of a diverse patient cohort reveals a conserved architecture in triple-negative breast cancer. Cancer Res.

[8] Bianchini G, De Angelis C, Licata L, Gianni L (2022). Treatment landscape of triple-negative breast cancer—expanded options, evolving needs. Nat Rev Clin Oncol.

[9] Shiao SL, Gouin KH, Ing N, Ho A, Basho R, Shah A, Mebane RH, Zitser D, Martinez A, Mevises NY, Ben-Cheikh B, Henson R, Mita M, McAndrew P, Karlan S, Giuliano A, Chung A, Amersi F, Dang C, Richardson H, Shon W, Dadmanesh F, Burnison M, Mirhadi A, Zumsteg ZS, Choi R, Davis M, Lee J, Rollins D, Martin C, Khameneh NH, McArthur H, Knott SRV (2024). Single-cell and spatial profiling identify three response trajectories to pembrolizumab and radiation therapy in triple negative breast cancer. Cancer Cell.

[10] Samantasinghar A, Sunildutt NP, Ahmed F, Soomro AM, Salih ARC, Parihar P, Memon FH, Kim KH, Kang IS, Choi KH (2023). A comprehensive review of key factors affecting the efficacy of antibody drug conjugate. Biomed Pharmacother.

[11] Paraskevopoulou MD, Hatzigeorgiou AG (2016). Analyzing MiRNA-LncRNA interactions. Methods Mol Biol.

[12] Liu J, Zhang M, Sun Q, Qin X, Gao T, Xu Y, Han S, Zhang Y, Guo Z (2023). Construction of a novel MPT-driven necrosis-related lncRNAs signature for prognosis prediction in laryngeal squamous cell carcinoma. Environ Sci Pollut Res Int.

[13] Xu Z, Zhang M, Guo Z, Chen L, Yang X, Li X, Liang Q, Tang Y, Liu J (2023). Stemness-related lncRNAs signature as a biologic prognostic model for head and neck squamous cell carcinoma. Apoptosis.

[14] Zhang M, Wang F, Xiang Z, Huang T, Zhou WB (2020). LncRNA XIST promotes chemoresistance of breast cancer cells to doxorubicin by sponging miR-200c-3p to upregulate ANLN. Clin Exp Pharmacol Physiol.

[15] Taft RJ, Pang KC, Mercer TR, Dinger M, Mattick JS (2010). Non-coding RNAs: regulators of disease. J Pathol.

[16] Zuo Y, Li Y, Zhou Z, Ma M, Fu K (2017). Long non-coding RNA MALAT1 promotes proliferation and invasion via targeting miR-129-5p in triple-negative breast cancer. Biomed Pharmacother.

[17] Wang L, Liu D, Wu X, Zeng Y, Li L, Hou Y, Li W, Liu Z (2018). Long non-coding RNA (LncRNA) RMST in triple-negative breast cancer (TNBC): expression analysis and biological roles research. J Cell Physiol.

[18] Niu L, Fan Q, Yan M, Wang L (2019). Biosci Rep.

[19] Chen FY, Zhou ZY, Zhang KJ, Pang J, Wang SM (2020). Long non-coding RNA MIR100HG promotes the migration, invasion and proliferation of triple-negative breast cancer cells by targeting the miR-5590-3p/OTX1 axis. Cancer Cell Int.

[20] Zhang G, Gao L, Zhang J, Wang R, Wei X (2023). Long non-coding RNA PTCSC3 suppresses triple-negative breast cancer by downregulating long non-coding RNA MIR100HG. Oncol Lett.

[21] Wang L, Luan T, Zhou S, Lin J, Yang Y, Liu W, Tong X, Jiang W (2019). LncRNA HCP5 promotes triple negative breast cancer progression as a ceRNA to regulate BIRC3 by sponging miR-219a-5p. Cancer Med.

[22] Luo N, Zhang K, Li X, Hu Y (2020). ZEB1 induced-upregulation of long noncoding RNA ZEB1-AS1 facilitates the progression of triple negative breast cancer by binding with ELAVL1 to maintain the stability of ZEB1 mRNA. J Cell Biochem.

[23] Castro-Oropeza R, Melendez-Zajgla J, Maldonado V, Vazquez-Santillan K (2018). The emerging role of lncRNAs in the regulation of cancer stem cells. Cell Oncol.

[24] McCabe EM, Rasmussen TP (2021). lncRNA involvement in cancer stem cell function and epithelial-mesenchymal transitions. Semin Cancer Biol.

[25] He Y, Jiang X, Duan L, Xiong Q, Yuan Y, Liu P, Jiang L, Shen Q, Zhao S, Yang C, Chen Y (2021). LncRNA PKMYT1AR promotes cancer stem cell maintenance in non-small cell lung cancer via activating Wnt signaling pathway. Mol Cancer.

[26] Li Y, Wang W, Wu M, Zhu P, Zhou Z, Gong Y, Gu Y (2022). LncRNA LINC01315 silencing modulates cancer stem cell properties and epithelial-to-mesenchymal transition in colorectal cancer via miR-484/DLK1 axis. Cell Cycle.

[27] Cruickshank BM, Wasson MD, Brown JM, Fernando W, Venkatesh J, Walker OL, Morales-Quintanilla F, Dahn ML, Vidovic D, Dean CA, VanIderstine C, Dellaire G, Marcato P (2021). LncRNA PART1 promotes proliferation and migration, is associated with cancer stem cells, and alters the miRNA landscape in triple-negative breast cancer. Cancers.

[28] Malta TM, Sokolov A, Gentles AJ, Burzykowski T, Poisson L, Weinstein JN, Kaminska B, Huelsken J, Omberg L, Gevaert O, Colaprico A, Czerwinska P, Mazurek S, Mishra L, Heyn H, Krasnitz A, Godwin AK, Lazar AJ, Stuart JM, Hoadley KA, Laird PW, Noushmehr H, Wiznerowicz M (2018). Machine learning identifies stemness features associated with oncogenic dedifferentiation. Cell.

[29] Frankish A, Diekhans M, Ferreira AM, Johnson R, Jungreis I, Loveland J, Mudge JM, Sisu C, Wright J, Armstrong J, Barnes I, Berry A, Bignell A, Carbonell Sala S, Chrast J, Cunningham F, Di Domenico T, Donaldson S, Fiddes IT, García Girón C, Gonzalez JM, Grego T, Hardy M, Hourlier T, Hunt T, Izuogu OG, Lagarde J, Martin FJ, Martínez L, Mohanan S, Muir P, Navarro FCP, Parker A, Pei B, Pozo F, Ruffier M, Schmitt BM, Stapleton E, Suner MM, Sycheva I, Uszczynska-Ratajczak B, Xu J, Yates A, Zerbino D, Zhang Y, Aken B, Choudhary JS, Gerstein M, Guigó R, Hubbard TJP, Kellis M, Paten B, Reymond A, Tress ML, Flicek P (2019). GENCODE reference annotation for the human and mouse genomes. Nucleic Acids Res.

[30] Sun Q, Qin X, Zhao J, Gao T, Xu Y, Chen G, Bai G, Guo Z, Liu J (2022). Cuproptosis-related LncRNA signatures as a prognostic model for head and neck squamous cell carcinoma. Apoptosis.

[31] Ritchie ME, Phipson B, Wu D, Hu Y, Law CW, Shi W, Smyth GK (2015). limma powers differential expression analyses for RNA-sequencing and microarray studies. Nucleic Acids Res.

[32] Liu G, Xiong D, Che Z, Chen H, Jin W (2022). A novel inflammation-associated prognostic signature for clear cell renal cell carcinoma. Oncol Lett.

[33] Langfelder P, Horvath S (2008). WGCNA: an R package for weighted correlation network analysis. BMC Bioinformatics.

[34] Liu J, Zhao J, Xu J, Sun Q, Qin X, Chen G, Gao T, Bai G, Guo Z (2022). SPINK5 is a prognostic biomarker associated with the progression and prognosis of laryngeal squamous cell carcinoma identified by weighted gene co-expression network analysis. Evol Bioinforma.

[35] Kamarudin AN, Cox T, Kolamunnage-Dona R (2017). Time-dependent ROC curve analysis in medical research: current methods and applications. BMC Med Res Methodol.

[36] Chen B, Khodadoust MS, Liu CL, Newman AM, Alizadeh AA (2018). Profiling tumor infiltrating immune cells with CIBERSORT. Methods Mol Biol.

[37] Huang L, Sun F, Liu Z, Jin W, Zhang Y, Chen J, Zhong C, Liang W, Peng H (2023). Probing the potential of defense response-associated genes for predicting the progression, prognosis, and immune microenvironment of osteosarcoma. Cancers.

[38] Rooney MS, Shukla SA, Wu CJ, Getz G, Hacohen N (2015). Molecular and genetic properties of tumors associated with local immune cytolytic activity. Cell.

[39] Jiang P, Gu S, Pan D, Fu J, Sahu A, Hu X, Li Z, Traugh N, Bu X, Li B, Liu J, Freeman GJ, Brown MA, Wucherpfennig KW, Liu XS (2018). Signatures of T cell dysfunction and exclusion predict cancer immunotherapy response. Nat Med.

[40] Geeleher P, Cox N, Huang RS (2014). pRRophetic: an R package for prediction of clinical chemotherapeutic response from tumor gene expression levels. PLoS ONE.

[41] Ponti D, Costa A, Zaffaroni N, Pratesi G, Petrangolini G, Coradini D, Pilotti S, Pierotti MA, Daidone MG (2005). Isolation and in vitro propagation of tumorigenic breast cancer cells with stem/progenitor cell properties. Cancer Res.

[42] Gennari A, Andre F, Barrios CH, Cortes J, de Azambuja E, DeMichele A, Dent R, Fenlon D, Gligorov J, Hurvitz SA, Im SA, Krug D, Kunz WG, Loi S, Penault-Llorca F, Ricke J, Robson M, Rugo HS, Saura C, Schmid P, Singer CF, Spanic T, Tolaney SM, Turner NC, Curigliano G, Loibl S, Paluch-Shimon S, Harbeck N (2021). ESMO clinical practice guideline for the diagnosis, staging and treatment of patients with metastatic breast cancer. Ann Oncol.

[43] Schunemann HJ, Lerda D, Dimitrova N, Alonso-Coello P, Grawingholt A, Quinn C, Follmann M, Mansel R, Sardanelli F, Rossi PG, Lebeau A, Nystrom L, Broeders M, Ioannidou-Mouzaka L, Duffy SW, Borisch B, Fitzpatrick P, Hofvind S, Castells X, Giordano L, Warman S, Saz-Parkinson Z (2019). Methods for development of the European commission initiative on breast cancer guidelines: recommendations in the era of guideline transparency. Ann Intern Med.

[44] Hua Z, White J, Zhou J (2022). Cancer stem cells in TNBC. Semin Cancer Biol.

[45] Dean M, Fojo T, Bates S (2005). Tumour stem cells and drug resistance. Nat Rev Cancer.

[46] Visvader JE, Lindeman GJ (2008). Cancer stem cells in solid tumours: accumulating evidence and unresolved questions. Nat Rev Cancer.

[47] Alison MR, Lim SM, Nicholson LJ (2011). Cancer stem cells: problems for therapy?. J Pathol.

[48] Plaks V, Kong N, Werb Z (2015). The cancer stem cell niche: how essential is the niche in regulating stemness of tumor cells?. Cell Stem Cell.

[49] Gul S, Pang J, Yuan H, Chen Y, Yu Q, Wang H, Tang W (2023). Stemness signature and targeted therapeutic drugs identification for triple negative breast cancer. Sci Data.

[50] Pei J, Wang Y, Li Y (2020). Identification of key genes controlling breast cancer stem cell characteristics via stemness indices analysis. J Transl Med.

[51] Wouters MCA, Nelson BH (2018). Prognostic significance of tumor-infiltrating b cells and plasma cells in human cancer. Clin Cancer Res.

[52] Ahmed F, Kang IS, Kim KH, Asif A, Rahim CSA, Samantasinghar A, Memon FH, Choi KH (2023). Drug repurposing for viral cancers: a paradigm of machine learning, deep learning, and virtual screening-based approaches. J Med Virol.

[53] Ahmed F, Lee JW, Samantasinghar A, Kim YS, Kim KH, Kang IS, Memon FH, Lim JH, Choi KH (2022). SperoPredictor: an integrated machine learning and molecular docking-based drug repurposing framework with use case of COVID-19. Front Public Health.

[54] Ahmed F, Yang YJ, Samantasinghar A, Kim YW, Ko JB, Choi KH (2023). Network-based drug repurposing for HPV-associated cervical cancer. Comput Struct Biotechnol J.

[55] Ahmed F, Ho SG, Samantasinghar A, Memon FH, Rahim CSA, Soomro AM, Pratibha SN, Kim KH, Choi KH (2022). Drug repurposing in psoriasis, performed by reversal of disease-associated gene expression profiles. Comput Struct Biotechnol J.

[56] Liao C, Guo Y, Gong Y, Huang X, Liao X, Wang X, Ruan G, Gao F (2020). Clinical implications and nomogram prediction of long noncoding RNA FRGCA as diagnostic and prognostic indicators in colon adenocarcinoma. Medicine.

[57] Hegre SA, Samdal H, Klima A, Stovner EB, Norsett KG, Liabakk NB, Olsen LC, Chawla K, Aas PA, Saetrom P (2021). Joint changes in RNA, RNA polymerase II, and promoter activity through the cell cycle identify non-coding RNAs involved in proliferation. Sci Rep.

[58] Zhou X, Yao L, Zhou X, Cong R, Luan J, Wei X, Zhang X, Song N (2022). Pyroptosis-related lncRNA prognostic model for renal cancer contributes to immunodiagnosis and immunotherapy. Front Oncol.

